# Characterization and evaluation of the immobilized laccase enzyme potential in dye degradation via one factor and response surface methodology approaches

**DOI:** 10.1038/s41598-024-82310-0

**Published:** 2025-01-03

**Authors:** Abdelmageed M. Othman, Angelina G. Flaifil

**Affiliations:** 1 Microbial Chemistry Department, Biotechnology Research Institute, National Research Centre, Dokki, Giza, 12622 Egypt; 2 Faculty of Biotechnology, German International University, Regional Ring Road, East Cairo, New Administrative Capital, Cairo, Egypt

**Keywords:** Immobilization, Laccase, Degradation, Pollutants, Environmental biotechnology, Biochemistry, Biological techniques, Biotechnology, Chemical biology, Environmental sciences

## Abstract

In the current study, calcium alginate was used as a carrier for *Agaricus bisporus* CU13 laccase immobilization, with an immobilization yield of the entrapped laccase of 91.95%. Free and immobilized enzymes showed their best enzyme activity at 60 °C as an optimum temperature. Free laccase was nearly completely inactivated at high temperatures (70 and 80 °C C) after 30 min, whereas the immobilized form retained around 60 and 40% of its activity after 30 min at the same temperatures. The metal ion of MgSO_4_ showed the best impact on the *Agaricus bisporus* laccase activity with a relative activity of 113.1 and 106.8% at concentrations of 10 mM and 2.5 mM, respectively. The best Cibacron D-Blue SGL dye degradation by laccase enzyme was obtained at pH 6.0, 0.354 U of laccase, and 100 mg/L of dye using hydroxybenzotriazole (HBT; 1 mM) as a mediator. Optimization ramps obtained from the central composite design (CCD) approach indicate that the rate at which the laccase enzyme decolorizes dye has increased significantly when the concentrations of the enzyme, and HBT increased, whereas dye concentration decreased. Finally, the immobilized enzyme was found to be efficient in decolorizing Cibacron D-Blue SGL dye in the presence of HBT as a mediator for different cycles.

**Supplementary Information:**

The online version contains supplementary material available at 10.1038/s41598-024-82310-0.

## Introduction

Environmental pollution is having serious and long-lasting effects on the planet, and it is becoming worse every day, where it can be found in the air, water, soil, sound, or light, among other forms. These factors include the burning of fossil fuels, acid rain, oil spills, and the release of hazardous waste from businesses, such as plastics, heavy metals, and nitrates^1^. Every year, there is a serious hazard to both human health and the ecology from the overuse and discharge of dyes into soil and water, where more than 7 million tons of dyes are used worldwide^2^. The waste effluent from many sectors, including textile, paper, leather, and plastics, is the source of synthetic organic dyes, which are aromatic compounds that constitute a significant class of colored pollutants. These intricate organic substances have the potential to be poisonous, mutagenic, and cancer-causing^3,4^.

Reactive dye Cibacron D-Blue SGL is often used for dyeing and printing in textile and other industrial applications, and due to it is particularly recognized for its vivid blue hue, Cibacron D-Blue SGL is frequently applied to cellulose fibers, including rayon and cotton. The capacity of reactive dyes to create covalent connections with fibers is well recognized since it leads to great wash and light fastness. To prevent contaminating the environment, reactive dye-containing wastewater must be treated before being disposed of^5^. Wastewater from the textile industry is treated using a variety of techniques, such as chemical oxidation, phytoremediation, coagulation, photocatalytic degradation, membrane filtering, biodegradation, and enzymatic remediation. Using enzymes, either free or immobilized, is seen to be novel, sustainable, and effective when it comes to enzyme remediation, where laccases and peroxidases are the primary enzymes that break down dyes to mitigate their impact on the ecosystem^6^.

Laccases are increasingly used for industrial applications, especially as biocatalysts in the detoxification of wastes in bioremediation processes^3,7^. Numerous bacteria, fungi, and plants have the multi-copper oxidase laccase, where through generating extracellular ligninolytic enzymes like laccase, fungi have a potent potential to metabolize dyes. The paper industry, biofuel manufacturing, and environmental cleanup have all used laccase because of its high catalytic activity and low substrate specificity, where laccase can efficiently break down dyes, according to recent research^2^. Multicopper oxidases, or laccases, are metalloproteins that endogenously and exogenously oxidize a variety of phenolic and nonphenolic compounds. These enzymes catalyze a coupling process that transfers a scavenged electron from the substrate to molecular oxygen during substrate oxidation, which is then reduced to water^8^.

Many xenobiotic substances, including polycyclic aromatic hydrocarbons, chlorinated phenolics, and synthetic dyes can be oxidized by the laccase enzyme^9^. Researchers have published some data on laccase’s degradation of various reactive dyes, either by its degradation processes or by the product’s disappearance as determined by chromatographic or spectrophotometric techniques. Low stability, low productivity, and high manufacturing cost are the main constraints on laccase, which is employed in dye degradation. By immobilization, these cost-effective issues may be minimized and their economic worth raised^10^. Laccase can be immobilized using a range of polymeric support materials, such as alginate, silicate minerals, cellulose derivatives, chitosan, polyaniline, polyamide, and polyacrylic polymers^11^. An intriguing strategy with several noteworthy benefits is using immobilized laccase on calcium alginate. This can greatly improve the enzyme’s thermal and operational reusability and stability to work well for an extended time, which is essential for industrial applications that cut costs and minimize waste. By preserving ideal conditions for catalysis, the calcium alginate matrix offers the laccase a regulated microenvironment and may even increase its activity. Because calcium alginate is also non-toxic and biocompatible, it may be used in a variety of procedures, such as biotechnological and environmental remediation^12^.

The scarcity of marketed laccases, however, makes laccase-based biocatalytic procedures difficult to perform, especially, in difficult reaction circumstances, as in organic solvents, they exhibit broad substrate specificities and enzyme stabilities^13^. Furthermore, the development of biocatalytic processes is impeded by their poor production yield due to the high cost of producing enzymes^14^. They are also vulnerable to varying operating circumstances because to their short service life, reusability, and lack of long-term operational stiffness. Immobilization techniques including entrapping, cross-linking, adsorption, covalent bonding can be used to overcome these drawbacks^15^. By improving the qualities of enzymes, such as their resistance to severe temperatures and capacity to withstand high temperatures, the immobilization method is the most basic and effective technique to overcome these limitations^3,16^. Laccase’s protein structure is strengthened and stabilized by immobilization, which improves the enzyme’s functional characteristics. Furthermore, compared to free laccase, immobilized laccase is more reusable, which makes it a desirable substitute for conventional dye degradation methods and in practical water treatment applications^17^.

**Fig. 1:**
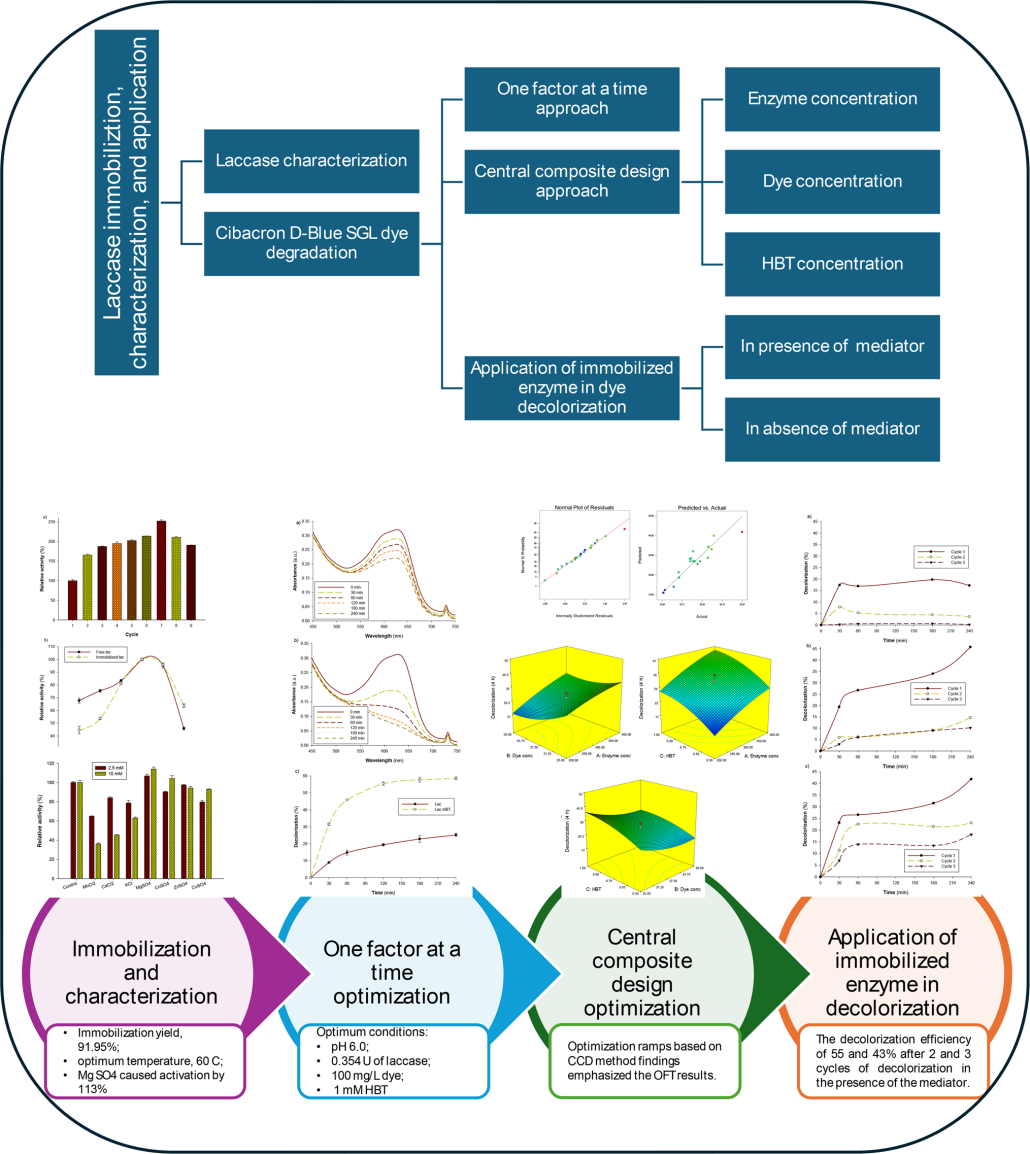
A flowchart visualizing the process steps regarding laccase immobilization, characterization, and application in Cibacron D-Blue SGL decolorization.

The objective of the current work is to increase laccase’s stability and reusability, which are frequently restricted in free form by environmental variables like pH and temperature. To enhance laccase’s efficacy in catalyzing processes, including dye degradation, over extended periods, alginate beads can create a protective matrix that stabilizes the enzyme. Compared to synthetic supports, the use of alginate, a biocompatible and biodegradable substance, minimizes environmental effects and is consistent with sustainable biocatalysis procedures. Furthermore, this study may investigate varied circumstances to maximize laccase activity, offering a flexible platform for diverse applications. For that, we aim throughout this work to (1) characterize the laccase of *Agaricus bisporus* CU13 and determine its potential for degradation of environmental pollutants; (2) ascertain the potential of fungal laccase from *Agaricus bisporus* CU13 for bioremediation of environmental pollutants using the Cibacron D-Blue SGL dye as a model; (3) employ response surface methodology to maximize laccase dye degradation capacity; and (4) apply the immobilized laccase in dye degradation cycles. A flowchart visualizing the process steps regarding laccase immobilization, characterization, and application in Cibacron D-Blue SGL decolorization is presented in Fig. 1.

## Materials and methods

### Chemicals

2,2´-azino-bis(3-ethylbenzothiazoline-6-sulphonic acid) (ABTS) and 1-hydroxybenzotriazole hydrate (HBT) were provided by Sigma-Aldrich (USA), whereas sodium alginate was ordered from Thermo Fisher Scientific (USA). Calcium chloride and bovine serum albumin (BSA) were obtained from Merck (Germany). The Textile Industries Institute, National Research Centre, Cairo, Egypt, provided Cibacron D-Blue SGL dye (Sigma, USA). Analytical grade chemicals were utilized in the investigation and straight out of the package without further purification.

### Enzyme source

The fermentation broth of the *Agaricus bisporus* CU13 fungal strain, which was obtained from the National Research Center, Cairo, was used as the crude laccase enzyme source. The culture conditions for the fungal strain were pH 7.0, 15.00 (g/L) soluble starch, 5.52 (g/L) yeast extract, and 24 days of incubation. Following the microbe’s culture, the broth was filtered, and the resulting filtrate was utilized as the source of the laccase enzyme^4^.

### Laccase enzyme activity and protein estimation

With the appropriately diluted enzyme sample in a total reaction volume of 2.0 ml, the enzyme activity of laccase was measured using 0.5 mL of 0.3 mM ABTS solution in citrate buffer (pH 4.5, 0.1 M) as substrate. We used a JASCO UV-Visible/NIR spectrophotometer to track the absorbance rise for one minute at 436 nm and laccase activity was assessed according to the following Eq. 1^8^:

1 $$Laccase\;activity\;\left( {\frac{U}{{ml}}} \right)=~\frac{{\Delta A~~ \times ~{V_{t~~~}} \times ~{{10}^6}}}{{\Delta t~ \times ~l~ \times ~\varepsilon ~ \times ~{V_s}~ \times ~1000}}$$

where Δ_*A*_ is the difference in absorbance, Δ_*t*_ is the period of incubation (min), *ε* is the extinction coefficient of ABTS (*ε*_436_ = 29,300 M^−1^ cm^− 1^), *l* is the cuvette diameter (1 cm), *V*_*t*_ is the total assay volume, and *V*_*s*_ is the enzyme sample volume. The ability of laccase to oxidize one µmol of ABTS per minute is defined as a unit of laccase.

To measure the immobilized enzyme activity, 1 g of immobilized laccase was combined with 3 mL of ABTS (0.3 mM) in citrate buffer (pH 4.5, 0.1 M). The enzyme units were computed using the accompanying Eq. 1^9^:

2 $$Laccase\;activity\;\left( {\frac{U}{{mg}}} \right)=\frac{{\Delta A~ \times ~{f_{dil}}~ \times ~{V_t}~ \times ~{{10}^6}}}{{\Delta t~ \times ~l~ \times ~\varepsilon ~ \times ~{m_{s~}} \times ~1000}}$$

where Δ*A* is the alteration in absorbance, *f*_*dil*_ is the dilution factor of the sample, *V*_*t*_ is the volume of the entire test, 106 is the exchange factor for concentration from M to µM, Δ*t* is the incubation duration in minutes, *l* is the diameter of the cuvette (1 cm), ε is the molar absorption coefficient of ABTS (*ε*_436_ = 29,300 M^−1^ cm^−1^), and *m*_*s*_ (mg) represents the immobilized enzyme’s mass. The amount of laccase required to oxidize one µmol of ABTS in a minute was designated as one unit (U).

Bradford method was employed to estimate the protein concentration utilizing Bovine Serum Albumin (BSA) as standard protein. BSA solution at an intensity of 0.1 mg/1 ml (100 µg/ml) was employed to get the standard curve of protein using serial dilutions of the BSA. Bradford reagent (0.2 ml) was added to the prepared concentrations (0.8 ml) to get a complete volume of 1 ml. The preparations were left 10 min at room temperature and the absorbance was determined at 595 nm.

### Enzyme immobilization

Thirty milliliters of Na-alginate (2%) were mixed with five milliliters of enzyme. To create Ca-alginate beads containing laccase, the mixture was gradually added drop by drop to calcium chloride (0.2 M) while being stirred. Before being used, the immobilized laccase in beads was removed from the solution and kept in potassium phosphate buffer pH 7.0 (0.1 M). Laccase activity and protein concentration at room temperature were measured in order to monitor the immobilization parameters. By comparing the units of enzyme extracted from the supernatant to the main enzyme activity using the following equation, the immobilization yield was computed:

$$Immobilization\;yield=({A_i} - {A_f})/{A_i} \times 100$$

where *Ai* constitutes the laccase’s initial activity, and *Af* is what’s left of the supernatant’s enzyme activity. By dividing the amount of protein that was removed from the supernatant by the carrier weight, protein loadings were computed.

$$Protein\;loading = \left( {P_{i} - Pf} \right)/W$$

where *Pi*, is the protein’s initial concentration (mg), *Pf*, is the ultimate concentration of protein, and W, the weight of the carrier (g)^18^.

### Operational stability

A reaction involving 1 g of the immobilized enzyme and 3.0 mL of substrate (0.3 mM ABTS) at room temperature and 50 rpm stirring force was used to assess the operational steadiness of the immobilized laccase. Samples were evaluated every minute over nine cycles of five minutes each. After removing the substrate at the conclusion of each cycle, the immobilized enzyme was cleaned three times with distilled water and then suspended again in a fresh buffered substrate solution. The control for relative activity (100%) was the ABTS oxidation activity of the first batch.

### Optimum temperature and thermal stability of laccase

The ideal laccase working temperature was estimated by tracking the enzyme activity at a range of temperatures from 30 ºC to 90 ºC while using conventional test conditions. In order to assess the thermal stability, the enzyme samples were incubated at 50 ºC, 60 ºC, 70 ºC, and 80 ºC without substrate in potassium phosphate buffer (pH 7, 0.1 M).

### Metal ions

Different mineral salts derived from different cations (NaCl, MnCl_2_, CaCl_2_, KCl, MgSO_4_, CaSO_4_, ZnSO_4_, CuSO_4_, FeSO_4_) were utilized to determine the enzyme activity activators and inhibitors. Two distinct amounts of each metal, 2.5 mM and 10 mM were applied to check the concentration effect of metal ions, whereas the metal salts were not added to the control sample. After a one-hour incubation period, the enzyme and metal ion-containing solutions were tested for laccase activity using reaction standard procedures.

### Decolorization of different dyes by *A. bisporus* laccase

An investigation was conducted employing the *A. bisporus* laccase to decolorize the following dyes: 2,6-dichlorophenol indophenol sodium salt D 5110, Cibacron D-Blue SGL, Methylene Blue (Basic), Lanasol, Maxilon Blue, and Acid dye Lanapel Red BM 143-PL. To achieve the desired concentrations, stock solutions of the dyes were made in distilled water. Both dye and laccase made up 1.2 milliliters of the buffered reaction mixture containing appropriate amounts of dye and laccase enzyme. After adding crude laccase, the reactions were started and left to incubate for different time intervals of up to 48 h at 30 °C. Subsequent to the decolorization initiation process, the JASCO UV-Visible/NIR spectrophotometer was utilized to measure the dyes’ absorption spectra between 350 and 800 nm.

### Optimization of dye decolorization

#### One variable per time optimization

##### Cibacron D-blue SGL dye decolorization as a function of enzyme concentration

**Fig. 2:**
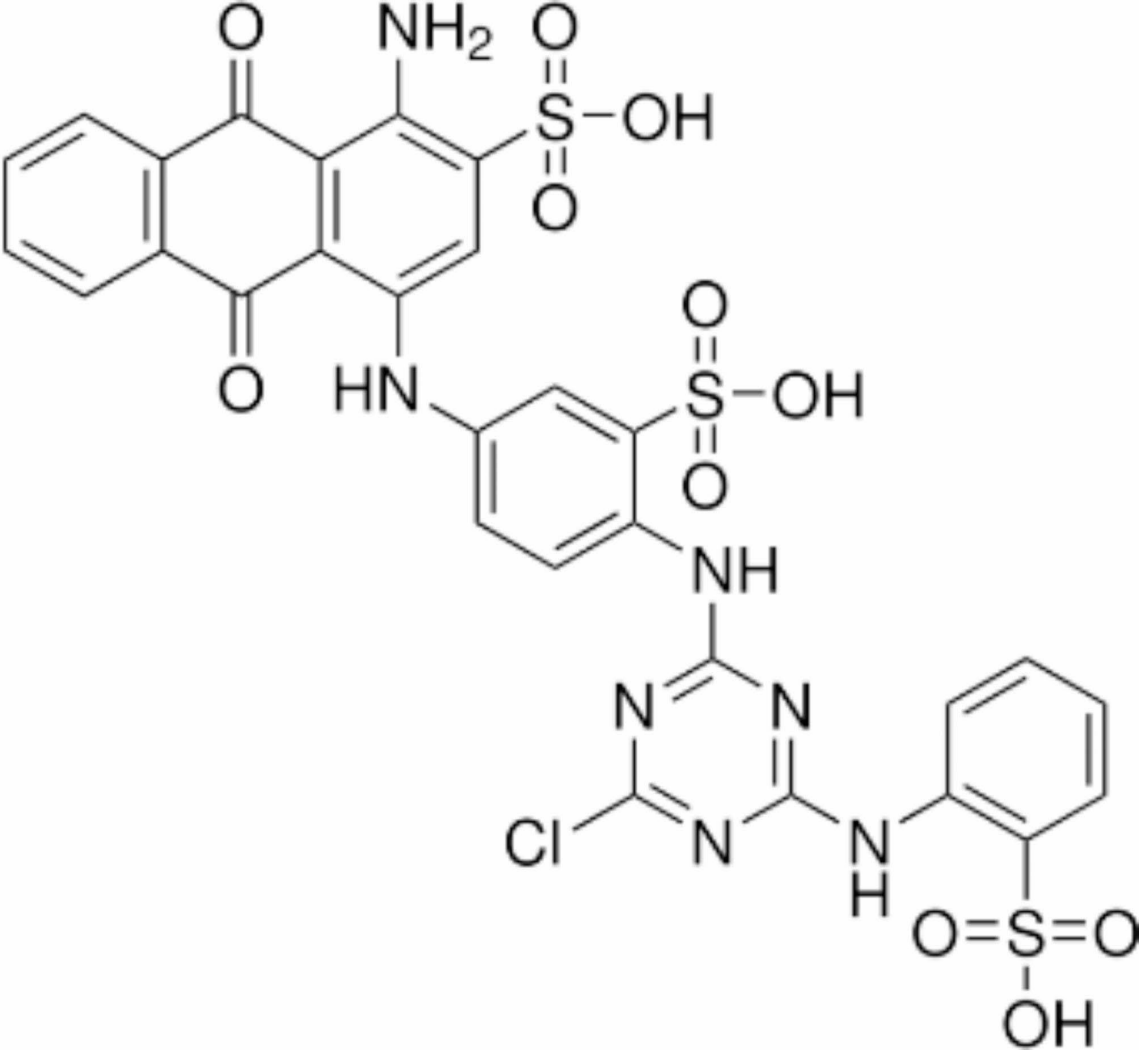
Cibacron D-Blue SGL structure.

The effectiveness of *A. bisporus* laccase in decolorizing Cibacron D-Blue SGL dye (Fig. 2) over other dyes led to the selection of this dye for further experiments. A complete reaction mixture of 1.2 ml containing the precise quantity of Cibacron D-Blue SGL dye (100 mg/l) and potassium phosphate buffer (pH 7.0, 0.1 M) was mixed with various quantities of laccase enzyme (0.018, 0.035, 0.071, 0.142, 0.354, and 0.708 U). For 0, 10, 30, 60, 120, 180, and 240 min at room temperature, the absorbance peak decrease between 800 and 350 nm was monitored.

##### Cibacron D-blue SGL dye’s decolorization in relation to its concentration

A complete reaction mixture of 1.2 ml containing the precise amount of enzyme (0.35 U) and potassium phosphate buffer (pH 7.0, 0.1 M) was mixed with several doses of Cibacron D-Blue SGL dye (25, 50, 100, 200, and 500 mg/l). For 0, 10, 30, 60, 120, 180, and 240 min at room temperature, the absorbance peak decrease between 800 and 350 nm was determined.

##### The impact of pH on the decolorization of Cibacron D-Blue SGL

The precise amount of *A. bisporus* laccase enzyme (0.35 U) and buffering systems (0.1 M) at pH values of 4.5, 6.0, 7.0, and 10 were combined with Cibacron D-Blue SGL dye (100 mg/l) in a total reaction mixture of 1.2 ml. The absorbance peak decrease between 800 and 350 nm was observed for 0, 10, 30, 60, 120, 180, and 240 min at room temperature.

##### HBT mediator systems’ effects on the decolorization of Cibacron D-Blue dye

Through the use of both free and immobilized laccase, the impact of the HBT mediator system on Cibacron D-Blue SGL dye decolorization was investigated. Regarding the free enzyme, 25 mg/l of dye was mixed with 0.35 U of the enzyme using potassium phosphate buffer at pH 6.0, either with or without 1 mM HBT, and incubated for 0, 10, 30, 60, 120, 180, and 240 min at room temperature, reaction mixtures were incubated. The impact was also examined by employing the immobilized enzyme in a reaction mixture (25 ml) including 5 g of the demobilized enzyme, 5 ml of potassium phosphate buffer, and 625 ml of dye in the presence or absence of 1 mM HBT. Reaction mixtures were incubated for 0, 10, 30, 60, 120, 180, and 240 min at 30 °C with shaking at 100 rpm. Enzyme-free alginate beads were used as controls.

#### Optimization via central composite design (CCD)

Using the central composite design (CCD), enzyme, dye, and HBT concentrations were used to maximize the process of Cibacron D-Blue SGL dye decolorization by *A. bisporus* laccase. The statistical potential of the decolorization process at five levels (−2, −1, 0, 1, 2) was conducted using CCD to investigate the relationships between the measured parameters, employing eighteen runs including four center points (Table 1). The software program Design Expert^®^ (Version 7.0.0) was used to examine the obtained data.

**Table 1:** Actual and predicted responses to the center composite design.

Run	(A) Enzyme conc. (µl)	(B) Dye conc. (µl)	(C) HBT conc. (µl)	Decolorization (%)	
				Actual value	Predicted value
1	200	25	50	24.09	26.40
2	300	17	75	54.87	46.95
3	300	38	75	29.78	32.01
4	300	38	75	31.90	32.01
5	200	25	100	31.13	37.06
6	132	38	75	21.71	19.11
7	300	38	117	38.30	33.78
8	400	25	100	41.10	45.02
9	200	50	50	16.70	15.99
10	300	38	75	34.48	32.01
11	300	38	75	31.10	32.01
12	300	59	75	30.07	33.46
13	468	38	75	40.01	38.07
14	400	50	50	33.30	30.58
15	400	50	100	38.49	39.39
16	300	38	33	17.43	17.41
17	200	50	100	24.28	23.72
18	400	25	50	29.51	33.28

## Results and discussion

### The immobilization and reusability of immobilized enzyme

Several ways allow laccase immobilization to have a substantial molecular influence on enzyme activity. Initially, immobilization frequently improves thermal and operational stability by stabilizing the structure of the enzyme by limiting its mobility. Second, when unfavorable orientations prevent substrate contact, the immobilization technique may affect the active site’s accessibility by blocking or changing it. This will lower enzyme activity. Third, immobilization may provide a microenvironment that influences the binding and diffusion of substrates^20^. The objective of the current work was to create a viable catalytic system for various applications, especially Cibacron D-Blue SGL decolorization through immobilizing *A. bisporus* CU13 laccase using calcium alginate as a carrier. Protein loading concentration and laccase immobilization yield (%) were used to gauge the success of the immobilization procedure, which involved entrapping laccase in calcium alginate. Although the protein loading was 0.022 mg protein/g support, the immobilization yield of the entrapped laccase was determined to be 91.95%, which declares the efficiency of immobilized protein. Multiple factors can influence the application of alginate as a support. For example, laccase was immobilized by Ratanapongleka and Punbut^21^, who used 1% and 5% w/v of alginate to entrap the enzyme with an immobilization yield of 89.01–93.29% ^21^.

**Fig. 3:**
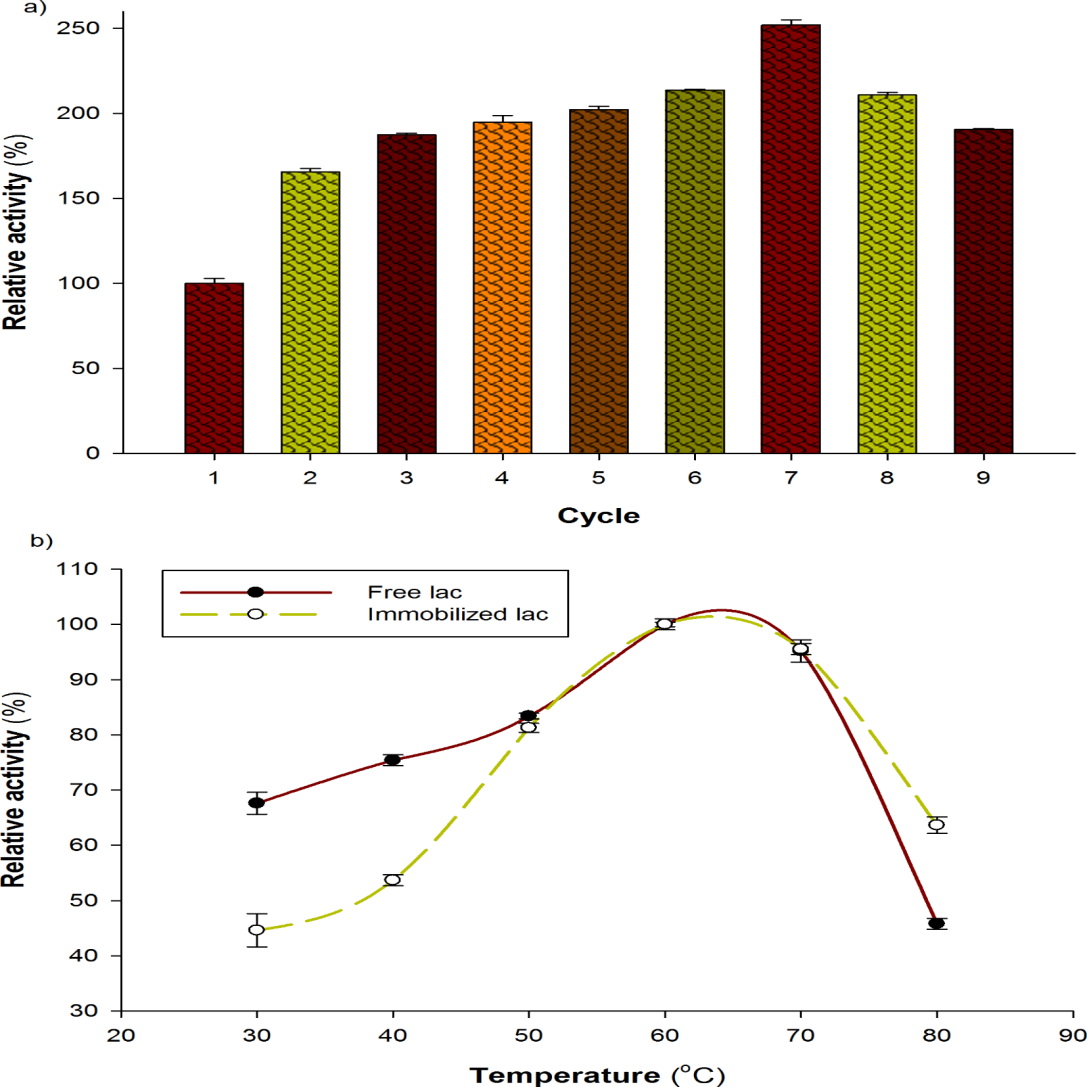
(**a**) Assessment of entrapped laccase reusability; 1 g of the entrapped immobilized laccase was reacted with 3.0 mL of ABTS (0.58 mM) in 100 mM citrate buffer (pH 4.5) at 25 ºC and 50 rpm for 9 cycles. (**b**) Effect of temperature on laccase activity articulated as relative activity (%) for both free and entrapped laccase, using ABTS (0.5 mM) as a substrate for the enzyme assay.

One crucial aspect of immobilized enzymes is their capacity for reuse. Improved stability and reusability over the free enzyme can be expected by enzyme immobilization. Nine consecutive oxidative cycles were used to assess the laccase’s functioning durability after its entrapment in calcium alginate beads. According to our findings (Fig. 3a), the immobilized laccase may be employed again for several catalytic runs without experiencing a reduction in its catalytic occupancy (100% after 9 runs). Laccase from *Bacillus sp.* MSK-01 was immobilized in calcium alginate and subjected to many cycles of evaluation. It has been demonstrated that the laccase continues to keep its catalytic function after four cycles^22^. In another study after ten rounds of reuse, Patel et al.^23^ demonstrated that the enzyme maintained 93.1% of residual activity.

### Characterization of *A. bisporus* laccase

#### Optimal temperature

This experiment aimed to determine the ideal temperature for both free and immobilized *A. bisporus* CU13 laccase activity. The range of temperatures at which laccase activity was measured for the free and immobilized enzymes was 30 to 80 °C. At 60 °C, both enzymes displayed their maximum levels of activity. Because free laccase may withstand conformational changes when exposed to varying temperatures, it is less stable than immobilized laccase.

The data presented in Fig. 3b indicates that there was a progressive rise in free laccase activity as the reaction temperature was raised. Specifically, at temperatures of 30 °C, 40 °C, and 50 °C, the relative activities were around 67.6, 75.3, and 83.4%, respectively. The maximum laccase activity value was reached at 60 °C. The enzyme exhibited relative activities of 95.1% and 45.7% at temperatures of 70 °C and 80 °C, respectively, and a steady drop-in activity was seen when the temperature was raised above this point.

Regarding immobilized laccase, the region prior to the optimal temperature showed a lower relative activity compared to the free enzyme since the immobilized form needs more activation energy; however, following the optimal temperature, the immobilized enzyme provided protection, resulting in a greater relative activity than the free enzyme. Figure 3b shows that the immobilized sample had a relative activity of 63.65% at 80 C, whereas the free sample had a relative activity of 45.8%.

It has been demonstrated that laccase activity varies from 30 to 80 °C depending on the microbe from which the enzyme is isolated. *Thermothelomyces thermophilus* laccase, for instance, demonstrated the highest activity at 70 °C^18^. *A. bisporus* CU13 isoenzymes shown in earlier investigations that the laccase’s activity rose to 55 °C, which was thought to be its ideal temperature. Comparing the two isoenzymes’ relative laccase activity to the optimal temperature, it was found that at 40 °C and 50 °C, respectively, the relative activity values were 74.93 and 66.31%, respectively^4^. According to Sondhi et al.^22^, the laccase from *Bacillus sp.* MSK-01 exhibited optimal activity at 75 °C for the free enzyme and 85 °C for the immobilized laccase.

#### Thermal stability

**Fig. 4:**
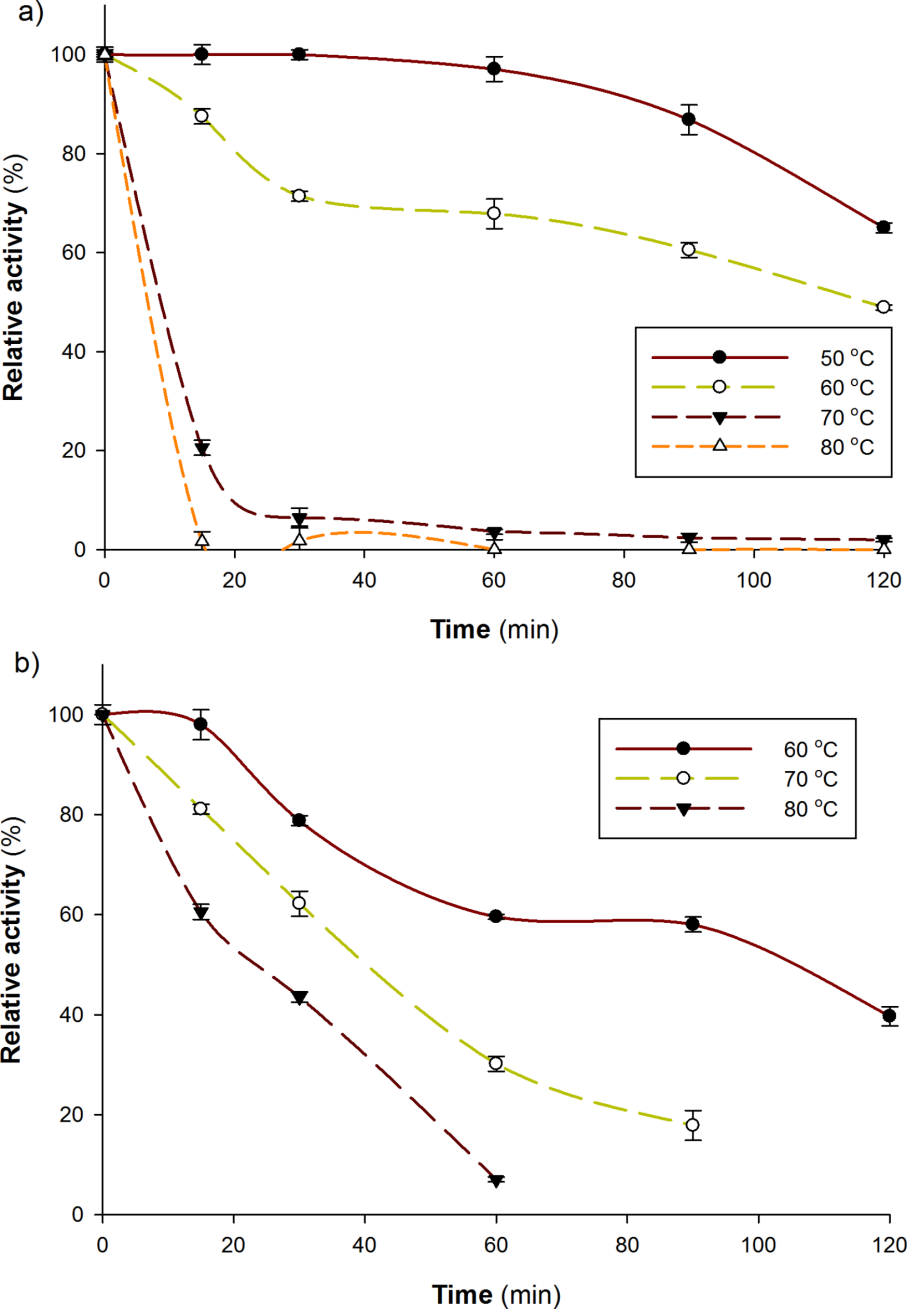
Thermal stability behavior of free (**a**) and immobilized (**b**) *A. bisporus* laccase; free or immobilized laccase were incubated in phosphate buffer (100 mM, pH 7.0) at 50, 60, 70, and 80 ºC. Samples were withdrawn at time intervals for 2 h and laccase activity was assessed using ABTS (0.58 mM) at 30 ºC and pH 4.5. All experiments were performed in duplicate, and the mean was represented.

Determining the temperature at which denaturation takes place, the characteristics of the denaturation peaks, and the enzyme’s overall stability across a range of thermal circumstances are all important factors to consider when analyzing thermal stability breakdowns. This knowledge is crucial for assessing the possible uses of enzymes in biocatalysis and industrial processes as it clarifies the limitations and robustness of these enzymes^24^. According to the data, the *A. bisporus* CU13 free laccase was able to stand at 50 °C for 60 min without showing any signs of losing activity. Additionally, the residual activity consistently declined with increasing exposure duration and temperature (Fig. 4). About 35, 51, 98, and 100% of the enzyme’s activity was lost after 120 min of incubation at 50, 60, 70, and 80 °C, respectively. The enzyme, however, displayed around 98.5% less activity after 15 min of incubation at 80 °C, suggesting that denaturation of the laccase was noticeable at higher temperatures. Based on the above-mentioned findings, we may deduce that the *A. bisporus* CU13 laccase exhibits thermal stability characteristics at moderate temperatures.

The findings demonstrated that free laccase was nearly completely inactivated at high temperatures (70 and 80 ºC) after 30 min, whereas the immobilized form retained around 60 and 40% of its activity after 30 min at the same temperatures (Fig. 4). It is believed that the protein’s enhanced atom vibration caused by high temperatures—which can break certain chemical bonds and significantly change the three-dimensional structure of proteins—is the cause of this expressive inactivation. At high temperatures, the enzymes change into new structures that prevent them from catalyzing.

Regarding the immobilized enzyme, the thermal stability of the enzyme at various temperatures was ascertained by measuring absorbance for five minutes at various periods. The immobilized laccase that is entrapped in calcium alginate expresses some increase in thermal stability. By examining a variety of temperatures, immobilized laccase exhibits better stability than the free enzyme. This may be because laccase is protected by the support when the temperature rises, which is clearer at high temperatures (70 and 80 °C). These results are opposite to the laccase immobilized on MWCNTs^25^ and TiO_2_-montmorillonite complexes^26^. We were unable to detect any enzymatic activity in the buffer solution in which the immobilized enzyme was incubated, suggesting that the inactive enzyme either remains adsorbed on the support or becomes inactive when released into the solution.

#### Metal ions

**Fig. 5:**
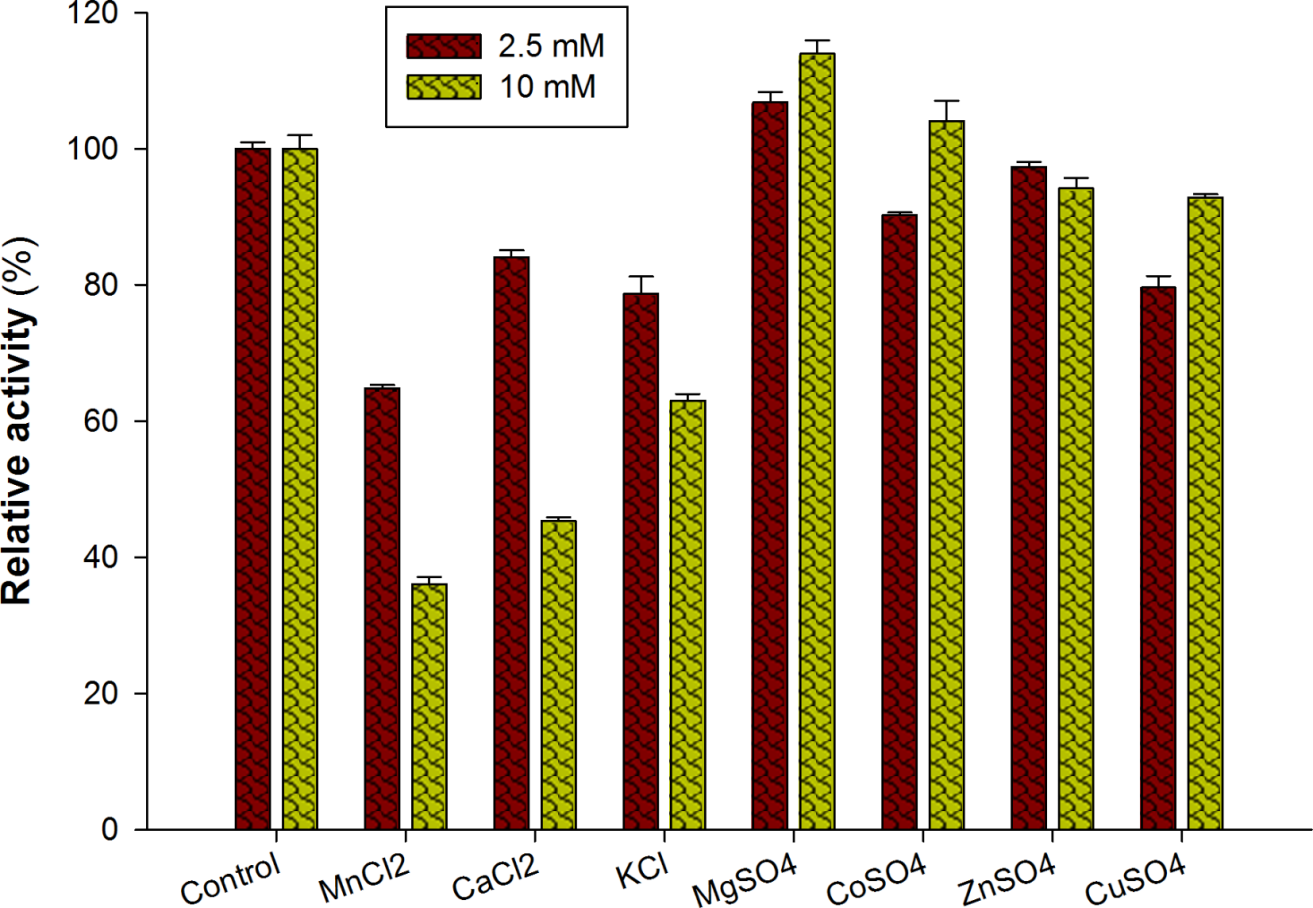
Effect of different metal salts on the *A. bisporus* CU13 laccase activity. Two distinct amounts of each metal, 2.5 mM and 10 mM were applied, and after a one-hour incubation period, the enzyme and metal ion-containing solutions were tested for laccase activity using reaction standard procedures.

One crucial element of the kinetics and operation of enzymes is the impact of metal ions on laccase activity. Multiple copper oxidases such as laccase are susceptible to the action of different metal ions, which can either increase or decrease their activity. Laccase activity depends on certain metal ions, such as copper, which function as cofactors. They play a crucial role in the redox processes that enable electron transport and are essential to the active site of the enzyme^27^. The effects of two distinct doses (2.5 mM and 10 mM) of the various metal ions (NaCl, MnCl_2_, CaCl_2_, KCl, MgSO_4_, CoSO_4_, ZnSO_4_, and CuSO_4_) on the laccase enzyme were investigated. With a relative activity of 113.1 at 10 mM and 106.8% at 2.5 mM doses of MgSO_4_ respectively, the laccase of *A. bisporus* yielded the greatest results regarding its effect on enzyme activity. However, MnCl_2_ (with a relative activity of 36% at 10 mM) showed the greatest inhibition of the investigated enzyme. Moreover, the least amount of influence on the enzyme activity was shown by CoSO_4_, ZnSO_4_, and CuSO_4_ (Fig. 5).

Analogously, two laccase isoenzymes of *A. bisporus* purified laccase were subjected to concentrations of 1 × 10^− 2^ M metal ions (Na^+^, Mn^2+^, Hg^2+^, Ca^2+^, K^+^, Mg^2+^, Co^2+^, Zn^2+^, Cu^2+^, or Fe^2+^). *A. bisporus* Lacc1 was significantly inhibited by Mn^2+^, Hg^2+^, Co^2+^, or Fe^2+^ in varying amounts, resulting in 58.96, 5.80, 83.55, and 1.00% of its original activity. The results indicated that Lacc 1 isoenzyme activity was boosted by Cu^2+^ to produce 138% of the activity without metal addition. According to Othman et al.^28^, Hg^2+^, Co^2+^, or Fe^2+^ also produced a notable suppression of *A. bisporus* Lacc2 activity, resulting in 0.85, 18.85, or 0.4% of the original enzyme activity. The degree to which metal ions contribute to electron transfer or stabilize the conformation of the enzyme can increase or decrease laccase activity. This impact is dependent on concentration and the enzyme variation. At low concentrations, some metal ions can increase activity; but, at greater quantities, enzyme inhibition may occur. Competitive binding at the active site or structural alterations that impact the stability of the enzyme may be the cause of this^29^. Reduced catalytic efficiency can result from toxic metals binding to the enzyme and altering its structure and activity. Metal ions can impact substrate binding and turnover rates by competing with the enzyme’s natural copper ions or by changing the electrical environment of the active site. Certain metal ions can bind at locations other than the active site, which can cause conformational changes that can have a positive or negative effect on the activity of an enzyme^30^.

### Application of *A. bisporus* laccase in dye degradation

#### Decolorization of different dyes by *A. bisporus* laccase

Using the *A. bisporus* CU13 laccase, the decolorization of six dyes was studied: 2,6-dichlorophenol indophenol sodium salt D 5110; Cibacron D-Blue SGL; Methylene Blue (Basic); Lanasol; Maxilon Blue; and Acid dye Lanapel Red BM 143-PL. In all studied dye solutions, the color intensity was quite high before the enzymatic reaction. However, after 96 h, all dyes except Cibacron D-Blue SGL showed very little decolorization (data not shown). This indicates that the enzyme functioned at its peak on the Cibacron D-Blue SGL. To do this, in subsequent studies, we will evaluate the decolorization of Cibacron D-Blue SGL utilizing the enzyme that is the subject of our investigation.

#### Optimization of dye decolorization

##### One variable per time optimization

**Fig. 6:**
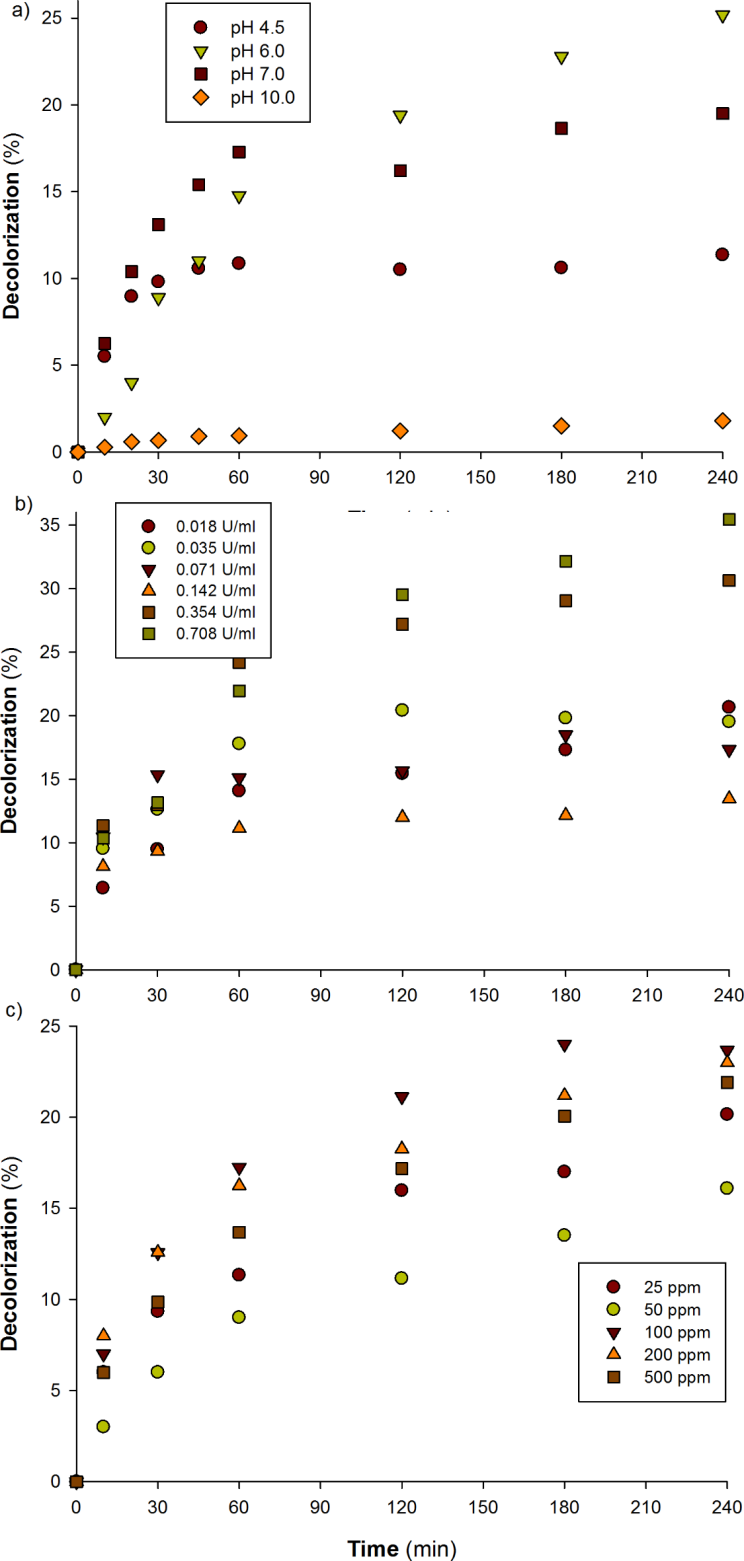
(**a**) Effect of pH value on Cibacron D-Blue SGL dye decolorization. The precise amount of laccase (0.35 U) and buffering systems (0.1 M) at pH values of 4.5, 6.0, 7.0, and 10 were combined with Cibacron D-Blue SGL dye (100 mg/l) in a total reaction mixture of 1.2 ml. (**b**) Effect of enzyme concentration on Cibacron D-Blue SGL dye decolorization. A precise quantity of Cibacron D-Blue SGL dye (100 mg/l) and potassium phosphate buffer (pH 7.0, 0.1 M) was mixed with various quantities of laccase enzyme (0.018, 0.035, 0.071, 0.142, 0.354, and 0.708 U). (**c**) Effect of dye concentration on Cibacron D-Blue SGL dye decolorization. Enzyme (0.35 U) and potassium phosphate buffer (pH 7.0, 0.1 M) were mixed with several doses of Cibacron D-Blue SGL dye (25, 50, 100, 200, and 500 mg/l). The absorbance peak decrease between 800 and 350 nm was observed for 0, 10, 30, 60, 120, 180, and 240 min at room temperature.

*The impact of pH level on the decolorization of Cibacron D-Blue SGL dye*. To identify the ideal pH range at which the enzyme may break down the dye, several pH buffers with pH values of 4.5, 6.0, 7.0, and 10.0 were employed in this experiment at room temperature (25 °C). The results indicated that pH 6.0 was the ideal pH for the laccase enzyme to break down dye. At pH 6.0, a notable decolorization rate was observed (Fig. 6a). Depending on the pH level, a progressive decline in the dyes’ rate of decolorization was seen with increases or decreases beyond this threshold. This may be explained by the redox potential value at various pH levels.

*Influence of enzyme concentration on the decolorization of Cibacron D-Blue SGL dye*. Various quantities of laccase enzyme (0.018, 0.035, 0.071, 0.142, 0.354, and 0.708 U) were used to find the ideal concentration for the dye’s decolorization. Following testing, it was discovered that the ideal activity for the degradation of Cibacron D-Blue SGL dye was 0.354 U. Only a little quantity of Cibacron D-Blue SGL dye degradation was increased by doubling the enzyme concentration (0.708 U), and this enhancement did not correspond with the utilized enzyme activity (Fig. 6b). For that, unless otherwise specified, we shall utilize a concentration of 0.354 U in all future studies.

*The impact of dye concentration on the decolorization of Cibacron D-Blue SGL dye*. The optimal dye concentration that can be degraded under typical circumstances was determined by evaluating several concentrations of Cibacron D-Blue SGL, which included 25, 50, 100, 200, and 500 mg/l. At 4 h (240 min), the optimal concentration was shown to be 100 mg/l. A progressive decline in the degradation percentage was noted upon raising the dye concentration over this threshold (Fig. 6c). Research conducted on Acid Blue dye by Othman et al.^28^ using two laccase isoenzymes revealed that *A. bisporus* CU13 purified laccase isoenzymes can decolorize the dye without the need for redox mediators. It was shown that after 30 min, Lacc1 could decolorize Acid Blue by 10.2, 19.0, 17.4, and 11.3% at doses of 25, 50, 100, and 200 mg/l. After sixty minutes, the percentages were 15.2, 25.1, 23.6, and 19.8%. The Acid Blue dye, on the other hand, could be decolored by 18.5, 27.2, 23.4, and 25.2%, respectively, at concentrations of 50, 100, 200, and 300 mg/l after 5 min using the *A. bisporus* Lacc2 isoenzyme. The decolorization percentage increased for the preceding concentrations at 30 min, reaching 33.3, 42.8, 36.1, and 34.1%, respectively^28^.

**Fig. 7:**
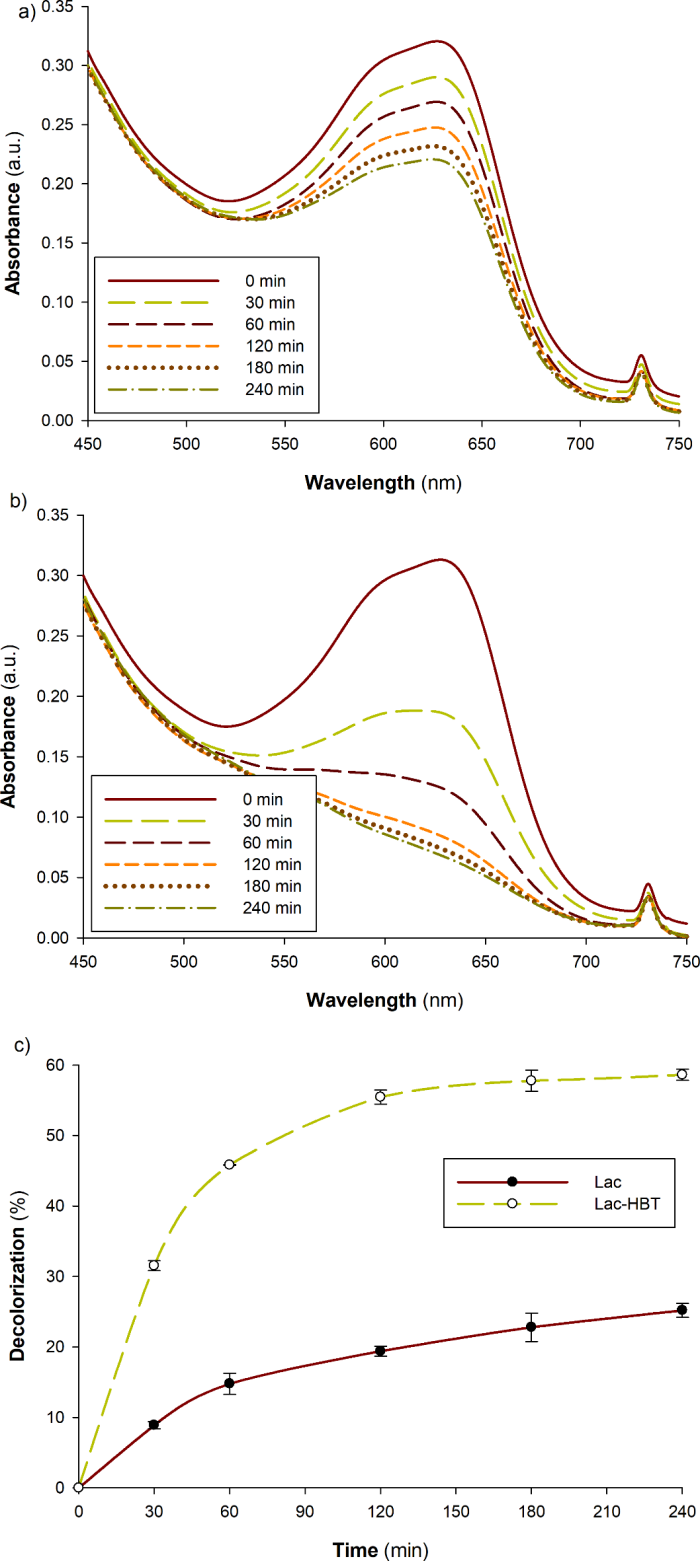
Effect of adding HBT mediator on Cibacron D-Blue SGL dye decolorization; (**a**) without HBT (laccase only) spectrum, (**b**) with HBT (laccase-mediator) spectrum, and (**c**) the decolorization percentage of both systems. 25 mg/l of dye was mixed with 0.35 U of the enzyme using potassium phosphate buffer at pH 6.0, either with or without 1 mM HBT, and incubated for 0, 10, 30, 60, 120, 180, and 240 min at room temperature.

*The impact of HBT mediators on the decolorization of Cibacron D-Blue SGL dye*. The addition of HBT mediator was used in this experiment to see under what circumstances the enzyme was most effective in breaking down the dye. The addition of HBT mediator was shown to significantly increase enzyme activity and improve Cibacron D-Blue SGL degradation, with a 60% decolorization percentage as opposed to 25% for laccase-HBT and laccase alone, respectively (Fig. 7a-c). In conclusion, it is evident that when laccase is combined with a mediator, less laccase may be required to have the same decolorizing effect. This would lead to a discernible preponderance in industrial application and a reduction in application costs. Commonly employed as an electron mediator, ABTS is efficient but can cause adverse reactions that lower its overall effectiveness. Methylene blue serves as both a substrate and a mediator in dye degradation experiments, which is why it is frequently utilized. Its usage may be constrained, nevertheless, by its toxicity and ability to suppress enzyme function^31^. Because of this, HBT was selected for this investigation because it offers the best possible combination of stability, reactivity, and compatibility with the enzyme systems involved in dye degradation. It is especially well-suited for applications focused on environmental cleanup because of its capacity to promote electron transfer without causing harmful side effects. HBT is a perfect mediator in this situation as it can also boost degradation rates due to its ability to increase enzyme activity^32^.

##### Central composite design (CCD) as a mean of optimization

Using the central composite design (CCD), the enzyme, dye, and HBT concentrations were used to maximize the process of dye decolorization by *A. bisporus* laccase. To investigate the statistical potential of the decolorization procedure at five levels (−2, −1, 0, 1, 2), CCD was used. To investigate the interactions between the examined factors, eighteen runs including four center points were carried out (Table 1). Design Expert^®^ (Version 7.0.0) Software was used to evaluate the collected data.

The use of several statistical techniques to identify and optimize the parameters most influencing the process and forecast their interactions to improve biological processes was established^33–35^. Because of their greater effectiveness in dye decolorization, the concentrations of enzyme, dye, and HBT were chosen to be examined using the CCD model, regarding the one factor per time optimization findings.

The CCD employed five levels (2, 1, 0, + 1, +2) from each characteristic parameter. Table 1 presents the results of the CCD experimental experiments, which were compared to the expected values. A quadratic model was used to understand the data and how they related to one another. The following equation, which links the response (dye decolorization percentage) to the CCD parameters (concentrations of enzyme (A), dye (B), and HBT (C)), is represented in terms of coded factors:

$$\begin{aligned} & {\text{Decolorization~}}\left( {\text{\% }} \right) = {\text{~}}32.01{\text{~}} + {\text{~}}5.64{\text{~A~}} - {\text{~}}4.01{\text{~B~}} + {\text{~}}4.87{\text{~C~}} + {\text{~}}1.93{\text{~AB~}} \\ & + {\text{~}}0.27{\text{~AC~}} - {\text{~}}0.73{\text{~BC~}} - {\text{~}}1.21{\text{~A}}2{\text{~}} + {\text{~}}2.90{\text{~B}}2{\text{~}} - {\text{~}}2.27{\text{~C}}2 \\ \end{aligned}$$

Where A, B, and C stand for the concentrations of the enzyme, dye, and HBT, respectively. The findings of the analysis of variance (ANOVA) are displayed in Table 2. The model is significant, as indicated by the model’s F-value of 5.67. A significant “Model F-value” has a 1.14% probability of occurring owing to noise. When “Prob > F” values are less than 0.05, it means that the model terms are significant. A, B, and C are important model terms in this instance. The model terms are not significant if the value is bigger than 0.10.

**Table 2:** Analysis of variance table (ANOVA) for response surface quadratic model of dye decolorization.

Source	Sum of squares	df	Mean Square	F Value	*p*-value Prob > F
Model	1251.23	9	139.03	5.67	0.011
A-Enzyme conc	434.03	1	434.03	17.71	0.003
B-Dye conc	219.62	1	219.62	8.96	0.017
C-HBT	323.79	1	323.79	13.21	0.007
AB	29.78	1	29.78	1.22	0.302
AC	0.58	1	0.58	0.02	0.881
BC	4.30	1	4.30	0.18	0.686
A^2^	18.46	1	18.46	0.75	0.411
B^2^	106.16	1	106.16	4.33	0.071
C^2^	65.05	1	65.05	2.65	0.142
Residual	196.05	8	24.51		
Lack of Fit	184.28	5	36.86	9.39	0.047
Pure Error	11.77	3	3.92		
Cor Total	1447.29	17			

R-Squared, 0.865; Adj R-Squared, 0.712; Adeq Precision, 8.391.

Model reduction might help your model if it has many unnecessary terms (apart from those needed to maintain hierarchy). The signal-to-noise ratio is measured using “Adeq Precision”, and it is preferred to have a ratio higher than 4.0. An appropriate signal is indicated by the ratio of 8.391, which suggests the design space can be navigated with the help of this model. Residual analysis was utilized to perform a diagnostic investigation of the created RSM for dye decolorization by laccase enzyme. More details about the constructed RSM model are provided by the residual analysis (Figure S1), where residual analysis against enzyme concentration, mediator concentration, and dye concentration was involved. To confirm the model’s assumptions, residual analysis is essential to the RSM test. The reliability of the selected model to the data is evaluated with the use of residual analysis, where a better fit may also be achieved by refining the model with the help of residual patterns.

**Fig. 8:**
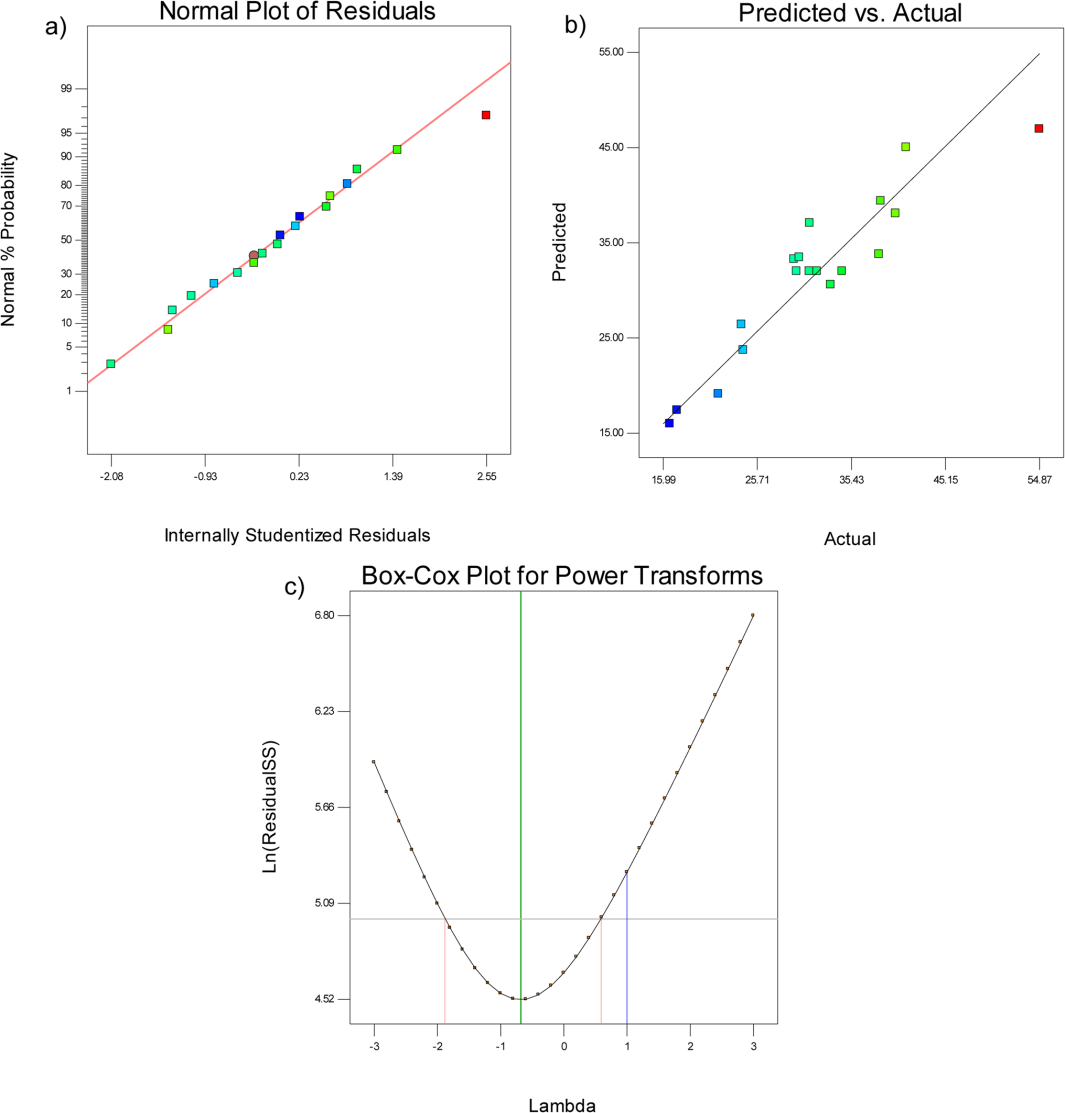
(**a**) The normal plot of residuals. (**b**) The relation between actual experimental values and model predicted values of dye decolorization. (**c**) The Box-Cox power transformation plot of the current quadratic model.

A normal probability plot was used to examine the effect of the residuals that influence the dye decolorization. Figure 8a illustrates the normal plot of residuals, which displays the residuals’ allocation. The dependability of the model is increased if the residuals show a normal distribution, which implies that the model’s assumptions are satisfied. Deviations from regularity might point to model misspecification and the need for modification or reworking. Verifying these presumptions contributes to the robustness and credibility of model’s predictions. Additionally, in the current study, results indicate that the residuals are normally distributed since a straight line connects all points in the normal plot of residuals versus the internally studentized residuals. Furthermore, this indicates that there is a strong agreement between the dye decolorization model projected values and the actual experimental results (Fig. 8b). The relationship between expected values and actual experimental results is critical to the validation of the model. It validates the model’s predictive power, highlights discrepancies that could indicate problems with the experimental setup, and aids in model refinement for optimization, enabling more accurate predictions in response surface methodologies. A statistical technique called the Box-Cox transformation is used to standardize and alter data in order to approximate a functional, acceptable, and trustworthy model^34^. When a model is fitted to transformed data, its goodness-of-fit is evaluated using metrics such as R-squared or adjusted R-squared. Additionally, residuals are examined for patterns or non-randomness to evaluate the model’s fit. These procedures are used in the Box-Cox transformation to validate the robustness and predictability of the model. Using the Box-Cox transformation, the residual normality of the quadratic model is improved by choosing an appropriate exponent (Lambda = λ). The power to which all data is intended to be increased is indicated by the Lambda value. Figure 8c shows the Box-Cox power transformation plot of the present quadratic model for dye decolorization optimization. This figure shows how power transformation affects the residuals’ normal distribution. Based on these findings, the constructed model may be used to determine the ideal laccase enzyme parameters for dye decolorization.

**Fig. 9:**
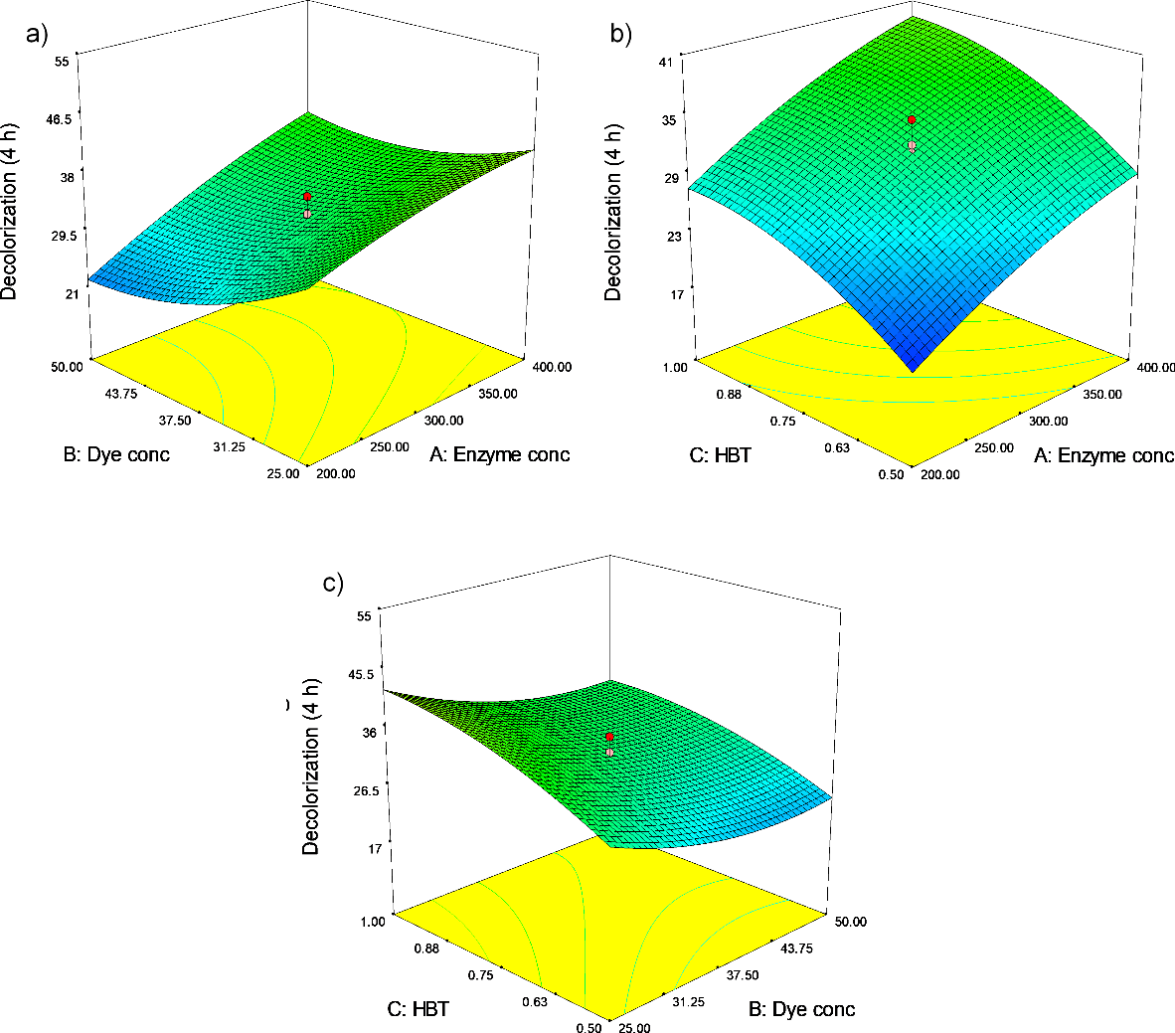
3D surface plots of dye decolorization via CCD approach. The presented results are the effect of the two active parameters whereas the other parameter is held at 0 levels (300 µl of enzyme, 38 µl of dye, and 75 µl of HBT).

The impact of the evaluated factors on the laccase enzyme’s ability to decolorize dye was illustrated using three-dimensional surface plots in Fig. 9 and counterplots in Fig. S2. While both types of plots are used to show how variables relate to one another, a contour plot condenses the data into a 2D representation with contour lines, which facilitates rapid comparisons and evaluations, while a 3D surface plot provides a more immersive view of the data’s shape and trends. For clarity and informational impact, we have included both of them here (Fig. 9 & Fig. S2). Two of the three parameters were examined at various interconnect values to show the graphs, while the third parameter remained fixed at its midpoint. For example, Fig. 9 shows the impact of interaction between studied parameters. In Fig. 9a, dye concentration (B) and enzyme concentration (A) on laccase enzyme-induced dye decolorization at a constant HBT concentration (75 µl) were explored. Based on all the data, the rate at which the laccase enzyme decolorizes dye has increased significantly when the concentrations of the enzyme (A), and HBT (C) increased, whereas dye concentration (B) has decreased (Fig. 9a–c). A reaction mixture comprising 25 µl dye and 100 µl HBT should be mixed with 0.4 mL laccase enzyme according to optimization ramps based on CCD method findings, which suggest a 45% decolorization rate (Fig. S3). Desirability ramps offer a framework for assessing various goals and assisting in the decision-making process, while three-dimensional plots give a visual depiction of the data that improves comprehension of correlations and patterns.

#### Immobilized laccase-induced decolorization of cibacron D-blue SGL

It is challenging to break down a pollutant like Cibacron D-Blue SGL using standard techniques since it is a resistant dye. Laccase’s capacity to oxidize dye molecules and break them down into less toxic, simpler chemicals make it a possible substitute for traditional techniques. The enzymatic decolorization method is eco-friendly, environmentally benign, and free of hazardous intermediates that are frequently created by chemical treatments^36^. In this study, laccase is rendered more stable, reusable, and easier to use in continuous operations due to its encapsulation in alginate beads to be more efficient in its bioremediation role. The oxidative processes that produce radical intermediates, which can then polymerize and break down further into simpler molecules, are the mechanisms by which laccase breaks down Cibacron D-Blue SGL dye. Numerous variables, such as pH, the concentration of the enzymes, and the presence of additional substrates, affect the precise nature of the degradation products^5^. Derivatives of the dye that have been hydroxylated or demethylated are examples of intermediate intermediates. Carboxylic acids are also produced when aromatic structures break down and can either enter metabolic pathways or get mineralized. Furthermore, other phenolic compounds may be generated, some of which may still be poisonous but less so than the original dye, depending on the particular substrate interactions^37^.

**Fig. 10:**
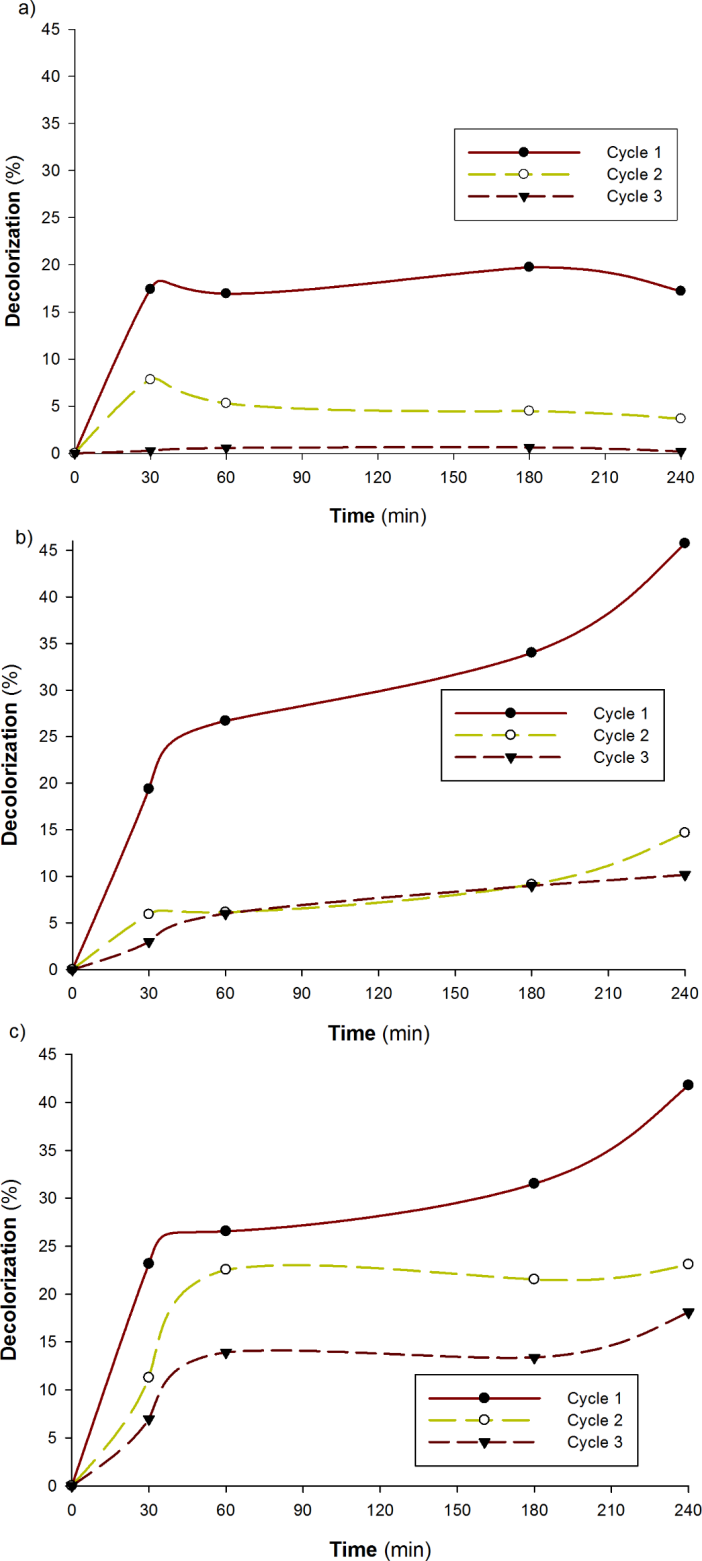
Decolorization of Cibacron D-Blue SGL application by immobilized laccase. (**a**) without enzyme and no HBT, (**b**) with enzyme and no HBT, and (**c**) with enzyme and HBT. The reaction mixture (25 ml) contained 5 g of beads (with or without enzyme), 625 µl of dye, and 1 ml of HBT at pH 6.0.

Regarding the immobilized enzyme application for decolorization of Cibacron D-Blue SGL, three samples—a control sample, a sample combined with the enzyme and HBT, and a sample without HBT—were used in the experiment, and each sample was analyzed for three cycles (Fig. 10a–c). The results obtained indicate the efficiency of using the immobilized enzyme for different cycles to decolorize Cibacron D-Blue SGL dye despite the decrease in decolorization efficiency due to the increase in cycle numbers. The data obtained from the recyclability of the immobilized enzyme in dye degradation shows that the efficiency of decolorization was reduced to 32 and 22% of its initial capability after 2 and 3 cycles respectively, of using the immobilized enzyme without mediator (HBT). In the case of introducing the HBT as a mediator for the decolorization process, the decolorization efficiency was increased to 55 and 43% of its initial capacity (in the first cycle) after 2 and 3 cycles of decolorization, respectively, which indicates the effective role of the mediation process. The results also indicate the absorbance of a small amount of the dye in the encapsulation beads as indicated by the control which agrees with Dominguez et al.^38^. Additionally, it was demonstrated that the addition of an HBT mediator produced the best results in comparison with the action of the enzyme only in the absence of a mediator role. In this regard, the research employed mediators to assist degrade industrial effluents in wastewater using laccase from *Bacillus sp.* MSK-01 using immobilized laccase mediator systems in Cu-alginate beads. Laccase and (1 mM) ABTS were immobilized in a Cu-alginate bead^22^. Table 3 represents a comparison between the current study laccase decolorization potential and some previous findings. Both free and immobilized enzymes are affected significantly by kinetic constants (Km and Vmax) values in the context of dye degradation, where a lower Km denotes a stronger affinity for the dye substrate. Lower Km values indicate improved binding, which increases the effectiveness of immobilized enzymes at low dye concentrations. Higher Km values, on the other hand, could reduce their efficacy in settings where substrate supply is limited. Furthermore, a larger Vmax denotes a stronger dye degradation rate at maximum. Immobilized enzymes exhibit improved stability and performance over time if their Vmax values remain high, which is advantageous for industrial applications^39^.

**Table 3:** Comparison between the current study laccase decolorization potential and some previous findings.

Laccase source	Targeted dye	Decolorization parameters	Decolorization efficiency	Reference
*Trametes versicolor*	Direct blue	81%	Dye concentration, 40 ppm; temperature, 40 °C; pH 4.0; time, 24 h	^40^
*Fusarium oxysporum* HUIB02	Malachite green	> 90%	pH, 4.5; temperature, 40 °C; time, 24 h	^41^
*Bacillus licheniformis* NS2324	bromophenol blue, phenol red, methylene blue	85%,75%,99.28% respectively	Time, 4 h; temperature, 40 °C	^42^
*Bacillus thuringiensis*	Methylene blue	95%	Temperature, 30 °C; pH, 6.0; agitation, 140 rpm; NaCl, 10 g/L; inoculum (v/v), 4%; time, 12 h	^43^
*Bacillus sp.* NU2	Methyl Orange, Congo Red, Malachite Green, Remazol Brilliant Blue R, and Reactive Blue 4	87%, 70%, 65%, 63% and 51% respectively	Temperature, 30 °C; time, 1 h	^44^
*Agaricus bisporus* CU13	Cibacron D-Blue SGL	60% using free laccase; 55 and 43% after 2 and 3 cycles using immobilized laccase	pH, 6.0; dye conc., 100 mg/L; 100 mM HBT	Current study

## Conclusion

Recent days have seen an increase in pollutants around our ecosystem, endangering it. Because it exposes them to several dangerous diseases, it also has a substantial impact on the species that live on our planet. Dyes are one kind of pollution that is widely used and spreads everywhere and has several harmful and detrimental effects on the environment. Finding a biological remedy rather than a chemical one was essential as chemicals harm the ecosystem. Enzymes work wonders when it comes to breaking down dyes, which makes them a valuable instrument for cleaning environments. Because of this, employing enzymes to break down dyes is thought to be highly advantageous and has produced encouraging outcomes in the field of biotechnology. The evaluation’s conclusion highlights the potential of immobilized laccase enzymes as a practical and long-lasting tool for pollutant degradation, emphasizing their contribution to the advancement of environmental biotechnology for ecosystems that are healthier and cleaner. For them to be fully utilized in useful environmental applications, research and development must continue.

With an immobilization yield of 91.95%, *Agaricus bisporus* CU13 laccase was entrapped successfully in alginate. The properties of the immobilized form were enhanced clearly where it retained around 60% of its activity after 30 min at 70 °C. The metal ion of MgSO_4_ activated the *Agaricus bisporus* laccase by 113.1 at concentrations of 10 mM. The best Cibacron D-Blue SGL dye degradation by laccase enzyme was obtained at pH 6.0, 0.354 U of laccase, and 100 mg/L of dye using HBT (1 mM) as a mediator. Finally, the immobilized enzyme was found to be efficient in decolorizing Cibacron D-Blue SGL dye (55%) in the presence of HBT as a mediator for different cycles.

## Electronic supplementary material

Below is the link to the electronic supplementary material.

Supplementary Material 1

## Data Availability

The datasets used and/or analyzed during the current study are available from the corresponding author upon reasonable request.
