# Supplementary material for: Characterization and evaluation of the immobilized laccase enzyme potential in dye degradation via one factor and response surface methodology approaches
Source: Sci Rep. 2025 Jan 3;15:735. doi: 10.1038/s41598-024-82310-0 (PMC11699123; doi:10.1038/s41598-024-82310-0)
Supplement: Supplementary file 1 — Supplementary Material 1 [file 41598_2024_82310_MOESM1_ESM.docx]

Fig S1: The residual analysis

Fig. S2: counterplots of dye decolorization via CCD approach. The presented results are the effect of the two active parameters whereas the other parameter is held at 0 levels (300 µl of enzyme, 38 µl of dye, and 75 µl of HBT).

Fig S3: The desirability ramps and 3D plot for the optimization criteria
